# Tissue Screening and Diagnosis of Transthyretin Amyloid Cardiomyopathy

**DOI:** 10.31083/RCM50854

**Published:** 2026-06-29

**Authors:** Ryan Tang, Harsh Patolia, Neehal Shukla, Judah Rajendran, Michel Chedid El Helou, Arianne Agdamag, Jerry D. Estep, Andres Carmona Rubio, Mazen Hanna, Trejeeve Martyn

**Affiliations:** ^1^Department of Internal Medicine, Cleveland Clinic, Cleveland, OH 44195, USA; ^2^George and Linda Kaufman Center for Heart Failure and Recovery, Heart Vascular and Thoracic Institute, Cleveland Clinic, Cleveland, OH 44195, USA; ^3^Amyloidosis Center, Cleveland Clinic, Cleveland, OH 44195, USA; ^4^Department of Cardiovascular Medicine, Cleveland Clinic Florida, Weston, FL 33331, USA

**Keywords:** ATTR-CM, transthyretin amyloid cardiomyopathy, early diagnosis, screening

## Abstract

Given the multi-organ involvement of transthyretin (ATTR) amyloidosis, associated symptoms such as peripheral neuropathy, biceps tendon rupture, lumbar stenosis, and bilateral carpal tunnel syndrome can develop concurrently with cardiac disease or even precede the cardiac diagnosis by up to a decade. Screening of tissue obtained opportunistically during an otherwise indicated procedure or diagnostic test has shown promise for the early identification of nascent cardiac amyloidosis (CA) in patients who might otherwise remain undiagnosed for years. This review aims to: (1) briefly summarize the cardiac and non-cardiac manifestations of transthyretin cardiomyopathy (ATTR-CM) that may raise clinical suspicion for the disease; (2) discuss tissue-based screening within contemporary ATTR-CM diagnostic pathways; (3) describe the diagnostic yield and management challenges of tissue screening, with a focus on early detection; (4) examine the treatment and surveillance implications of tissue screening in both symptomatic and asymptomatic individuals. Multidisciplinary collaboration (*e*.*g*., partnerships between hand surgery centers and amyloid centers) and criteria-based screening of specimens obtained during noncardiac and cardiac surgery may offer an effective strategy for identifying patients earlier in the associated disease course. However, given the variable diagnostic yield, the significant anxiety associated with comprehensive evaluation for ATTR-CM, and the potentially high cost of treating CA with one of the three therapies approved by the Food and Drug Administration (FDA), further refinement of screening criteria and additional investigation into the impact of early treatment on disease trajectory and outcomes are needed.

## 1. Introduction

Cardiac amyloidosis (CA) is an infiltrative cardiomyopathy characterized by the extracellular deposition of misfolded protein fibrils within the myocardium, leading to progressive heart failure. The two principal subtypes of CA are transthyretin (ATTR) cardiomyopathy (ATTR-CM) and light chain (AL) cardiomyopathy (AL-CM). In ATTR-CM, the normally stable tetrameric form of TTR is destabilized, forming pathologic amyloid fibrils that deposit in the extracellular matrix of various organs, such as the myocardium and nerves [1]. In contrast, AL amyloidosis is a clonal plasma cell disorder driven by plasma cells producing unstable light chains prone to misfolding. Both ATTR and AL amyloid fibrils exhibit a pathognomonic green birefringence when stained with Congo red dye and viewed under polarized light microscopy [2,3]. Although their mechanisms differ, both cause myocardial thickening and lead to diastolic dysfunction, arrhythmias, and conduction abnormalities [1,3,4].

ATTR amyloidosis is classified into sporadic wtATTR (wild-type) and inherited vATTR (variant or hereditary). The hereditary form results from pathogenic *TTR* gene mutations inherited in an autosomal dominant pattern. There are over 150 variants identified to date, which can lead to a wide spectrum of clinical presentations. The most prevalent variant in the United States is the *Val122Ile *(*p.V142I*) mutation carried by 3–4% of African Americans and individuals of Afro-Caribbean descent, which is strongly associated with the development of a late-onset cardiomyopathy and polyneuropathy [5]. Worldwide prevalence of wtATTR is estimated to be about 200,000 to 300,000 individuals versus vATTR is estimated to be around 50,000 individuals [6].

While ATTR-CM was previously characterized as a rare disease, increased clinician awareness and adoption of non-invasive diagnostic imaging have enabled improvements in screening at-risk populations. Despite the heightened awareness, it remains a significant cause of cardiovascular morbidity and mortality due to missed or late diagnosis [7], and current therapies are most effective when initiated early in the disease course, given its progressive nature [8]. Additionally, the diagnosis of ATTR-CM is challenging due to its similarities with more familiar etiologies of cardiomyopathy in older adults such as hypertension, chronic kidney disease, atrial arrhythmia, and hypertrophic cardiomyopathy.

**Fig. 1.**
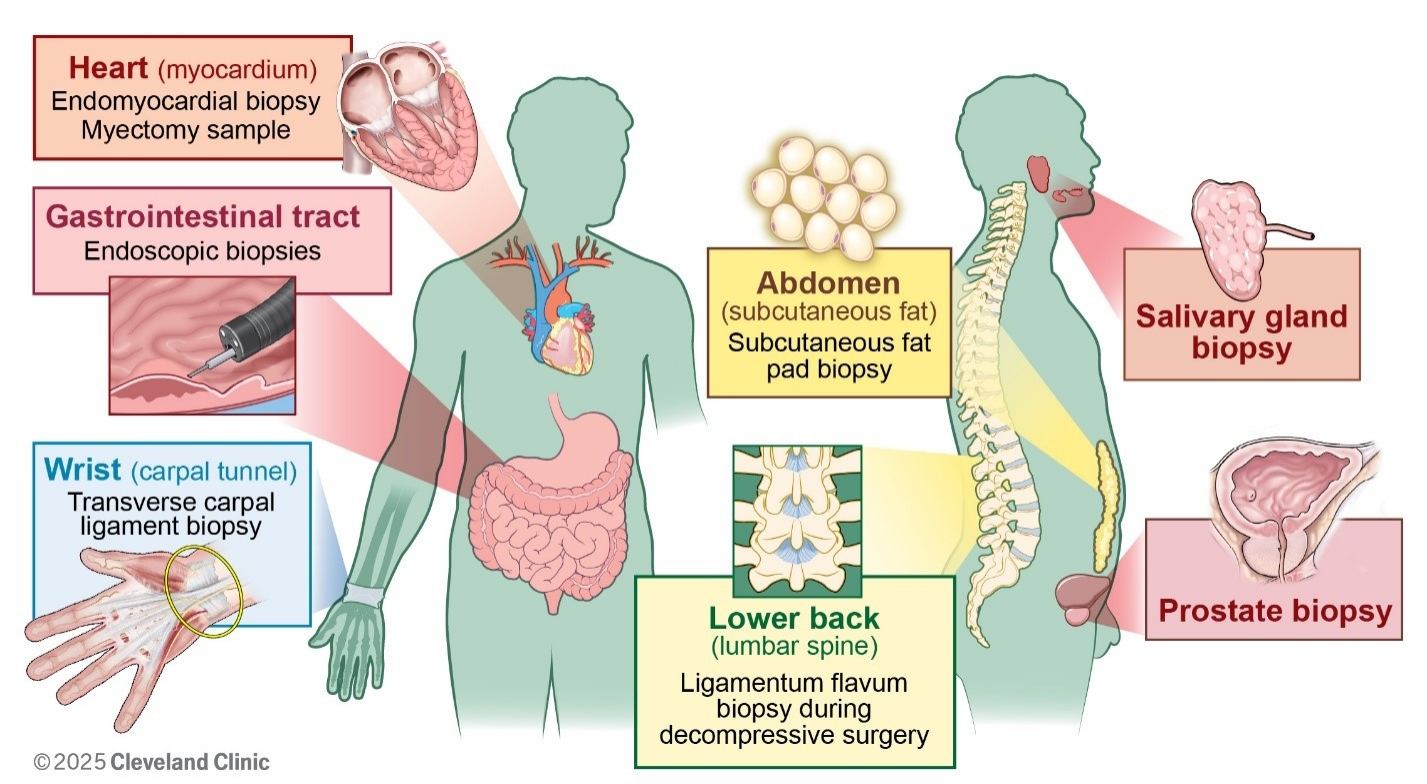
**Various sites for tissue biopsy to assist with the diagnosis of amyloidosis**. Some (like the heart, salivary glands, or subcutaneous fat) are targeted when clinical suspicion is high, whereas others may assist with the diagnosis of amyloidosis during other elective procedures.

Given the multi-organ involvement of ATTR amyloidosis, it is not uncommon for associated symptoms, such as peripheral neuropathy, lumbar stenosis, biceps tendon rupture, and bilateral carpal tunnel syndrome to develop concurrently or even pre-date the cardiac diagnosis by up to a decade (Fig. 1) [9]. Despite growing public awareness and the increasing prevalence of artificial intelligence (AI)-based tools for imaging interpretation, electrocardiogram (ECG)-based detection, risk scoring, and electronic health record review, the nuances of developing CA suspicion remain largely limited to a selected group of cardiologists and neurologists. In contrast, screening tissue obtained strategically—through otherwise indicated surgical procedures or diagnostic testing—has shown promise toward early identification of nascent CA in patients who might otherwise remain undiagnosed for many years.

This review aims to: (1) briefly review the cardiac and non-cardiac manifestations of ATTR-CM that may raise clinical suspicion for CA, (2) discuss tissue-based screening within contemporary ATTR-CM diagnostic pathways, (3) describe the diagnostic yield and management challenges of tissue screening, with a focus on early detection, and (4) discuss treatment and surveillance implications of tissue screening in both symptomatic and asymptomatic individuals. As there are now three therapies approved by the Food and Drug Administration (FDA) for ATTR-CM, the role of extra-cardiac and cardiac tissue-based diagnosis and treatment merits review [8].

## 2. Raising Clinical Suspicion: The Cardiac Presentation and Initial Workup

Patients with ATTR-CM most commonly seek medical attention due to symptoms of heart failure, arrhythmias, or conduction disease before ultimately receiving a diagnosis. Evaluation of a patient with these symptoms often starts with a thorough history, an echocardiogram, and an electrocardiogram.

In addition to cardiac manifestations, ATTR amyloid deposition can manifest as extra-cardiac symptoms, resulting in a multi-system disease that contributes to physical decline and frailty [1]. Common conditions associated with amyloidosis include bilateral carpal tunnel syndrome, lumbar spinal stenosis, peripheral neuropathy, and autonomic dysfunction that manifests as orthostasis or gastrointestinal (GI) symptoms or sexual dysfunction in males [1]. In addition to patient symptoms, a thorough family history is valuable in identifying the heritable subtypes of ATTR amyloidosis. Unexplained heart failure or neuropathy in several family members may prompt further suspicion for variant ATTR, though the median age of onset for many variants coincides with a “typical” age for incident heart failure (HF) in older adults.

Biomarkers such as high-sensitivity cardiac troponin and N-terminal pro-B-type natriuretic peptide (NT-proBNP) are elevated in patients with clinically significant ATTR-CM [10]. The National Amyloidosis Centre (NAC) staging system classifies patients into stages I, II, or III based on the NT-proBNP and estimated glomerular filtration rate (eGFR) and continues to be prognostic in the treatment era [11]. Unfortunately, even in this post-tafamidis (Pfizer Incorporated, New York, New York) treatment era, approximately half of patients are diagnosed with NAC stage II and III disease, the most severe stages. Marked elevation in NT-proBNP and declining renal function suggestive of advanced disease is still common in contemporary cohorts [12].

A transthoracic echocardiogram (TTE) of suspected patients with ATTR-CM can evaluate ventricular wall thickening due to the amyloid deposition. The degree of wall thickness suggestive of amyloidosis remains an ongoing point of discussion. While unexplained left ventricular wall thickness (LVWT) of greater than 12 millimeters (mm) along with heart failure in older adults should raise suspicion of infiltrative cardiomyopathy, there are a wide range of LVWT in those ultimately diagnosed with ATTR-CM [4]. The average LVWT at the time of trial enrollment in the seminal ATTR-ACT trial evaluating tafamidis versus placebo was 16–18 mm, suggesting late diagnosis and more advanced disease [13]. Encouragingly, patients enrolled in more contemporary trials like HELIOS-B and ATTRIBUTE-CM had lower median NT-proBNP and a lower proportion of New York Heart Association (NYHA) class III patients [14,15]. The constellation of features including ECG, echocardiographic, and multimodality imaging features are detailed in multiple other reviews and consensus documents and are not the focus of this review [2].

## 3. Common Invasive and Non-Invasive Diagnostic Techniques

Diagnostic workup of amyloidosis is well-established and begins with exclusion of AL amyloidosis using serum free light chains and serum and urine immunofixation followed by cardiac scintigraphy [2,16]. Technetium-99m bone-avid scintigraphy—most commonly using pyrophosphate (PYP) in the United States and 99mTc-3,3-diphosphono-1,2-propanodicarboxylic acid (DPD) or hydroxymethylene diphosphonate (HMDP) in Europe—enables visualization of myocardial amyloid deposition based on tracer uptake. A positive scan with grade 2 or 3 uptake confirmed by single-photon emission computed tomography (SPECT) imaging, combined with negative serum and urine immunofixation for monoclonal proteins and typical imaging features of cardiac amyloidosis is considered diagnostic for ATTR-CM [17].

Despite advancements in non-invasive techniques, endomyocardial biopsy (EMB) continues to play a role in select cases. Histologic examination with Congo red staining, demonstrating characteristic apple-green birefringence under polarized light, can confirm the presence of intracardiac amyloid deposits [2,18]. Additionally, mass spectrometry of specimens can differentiate ATTR from AL amyloidosis. In challenging cases, immuno-electron microscopy can specifically distinguish AL amyloidosis [19].

Although EMB provides high diagnostic accuracy, this technique is not without serious risks, such as cardiac perforation, tamponade, and arrhythmias. This procedure should be performed only at specialty centers supported by a multidisciplinary team of biopsy operators, advanced heart failure physicians, experienced pathologists with access to advanced techniques [20]. Thus, biopsy remains reserved for cases where non-invasive methods yield inconclusive results but clinical suspicion remains high, or when differentiating between amyloid subtypes is necessary for management. A common scenario in which EMB is performed is when a significant gammopathy is noted in the presence of a scintigraphy consistent with ATTR-CM.

Subcutaneous fat pad biopsy, abdominal fat aspiration, and salivary gland biopsy are less invasive approaches to amyloid detection. While these methods are useful for diagnosing extracardiac AL amyloidosis, the sensitivity is lower for ATTR amyloidosis [21]. A study of abdominal fat pad biopsies showed higher sensitivity than other modalities, noting a sensitivity of 91% [22]. One study of abdominal fat aspiration noted a 45.7% sensitivity of amyloidosis overall, with sensitivity as high as 83.8% for AL amyloidosis in particular [23]. An additional study evaluating the effectiveness of AL amyloidosis fat pad sampling noted a sensitivity of 76.6% [24]. For minor salivary gland biopsies, one study cited a sensitivity of 61.1% [25] while another study regarding labial salivary gland biopsy was reported to be 86.7% [26]. When combined with bone marrow biopsy or cardiac imaging, fat pad or salivary gland biopsy remain a valuable diagnostic tool if EMB is not feasible or in cases that require evaluation for systemic amyloidosis in which clinical acuity or ambiguity requires a stepwise approach [2,27]. While extracardiac biopsies are diagnostically valuable, cardiac involvement must be confirmed with nuclear scintigraphy or EMB. Guidance regarding surveillance of patients with confirmed extracardiac amyloidosis without signs of cardiac involvement remains unclear, though physicians should remain vigilant with consideration of routine surveillance with nuclear scintigraphy. In patients with advanced heart failure or aggressive light chain deposition, confirmation of extracardiac and cardiac involvement is crucial to facilitate initiation of therapy and consideration of advanced heart failure therapies.

Genetic screening remains crucial among those with a known family history of vATTR due to its autosomal dominant inheritance pattern. A diagnosis of vATTR in a family member should prompt cascade genetic screening among relatives of probands. Current European Society of Cardiology (ESC) guidelines recommend cardiac screening for these individuals known to be vATTR carriers starting at 10 years earlier than the young member of the family has developed amyloid cardiomyopathy. Though serum chemistries, TTE, and ECG are recommended yearly, nuclear scintigraphy is recommended every three years [2]. Despite variable penetrance, implementation of these practice guidelines led to effective detection of vATTR-CM, 25% of subclinical vATTR-CM now demonstrating any other hallmark findings of amyloid cardiomyopathy on ECG, TTE, or serum chemistries [28].

Overall, diagnosis of ATTR-CM requires a strategic, sequential approach that comprehensively excludes AL-CM. The high yield, invasive modalities are summarized in Table 1 (Ref. [22,23,25,26]), and the overall approach to the assessment when ATTR-CM is suspected is summarized elsewhere [2].

**Table 1.** **Deliberate sampling of common, conventional, and higher yield tissue biopsy sites when clinical suspicion is high**.

Screening modality	Yield	Notes
Endomyocardial biopsy	Near 100%	Gold standard diagnosis. Diagnostic means of testing for cardiac amyloidosis.
Subcutaneous fat biopsy/abdominal fat aspiration	15.5%–91%	Higher sensitivity (up to 91%) has been shown in fat pad biopsies than for abdominal fat aspiration, where vATTR demonstrates higher yield (45.1%) and wtATTR has lower yield (15.5%) [22,23].
Salivary gland biopsy	61.1%–86.7%	Minor salivary gland biopsy is of lower yield than labial salivary gland biopsy [25,26].

ATTR, transthyretin; vATTR, variant ATTR; wtATTR, wild-type ATTR.

## 4. Extra-Cardiac Manifestations of Transthyretin Amyloidosis

Recognizing early extracardiac signs of ATTR-CM is crucial, as they can serve as clinical “red flags” prompting further evaluation for amyloidosis before significant myocardial involvement occurs [29,30]. Among these manifestations, carpal tunnel syndrome, lumbar spinal stenosis, and gastrointestinal symptoms are the most common, frequently causing patients to interact with orthopedists, neurologists, or gastroenterologists long before an amyloidosis specialist. Among cardiologists outside of specialty amyloidosis centers, general awareness may be lower, which may lead to later referral [31]. Because amyloid deposition in these tissues may predate the onset of heart failure, these manifestations offer a unique window for early tissue-based diagnosis, which can prompt evaluation for cardiac involvement. The following sections review how these specific extra-cardiac manifestations have been leveraged for tissue-based evaluation of ATTR-CM (summarized in Table 2, Ref. [29,32,33,34,35,36,37,38]).

**Table 2.** **Yield of tissue screening during elective procedures that can serve as additional opportunities to diagnose cardiac amyloidosis**.

Screening modality	Yield	Notes
Myectomy sample	0.8%	27/3292 of patients with cardiac amyloidosis on analysis of myectomy specimens [37].
Gastrointestinal (GI) biopsy	Variable	Yield varies widely based on biopsy site and type of amyloidosis. One prospective evaluation of 181 samples yielded no positive samples on Congo red staining [33,35]. Some studies report duodenal biopsy positivity in up to 100% of patients, others estimating 50–70%; similarly, rectal biopsy was previously a sensitive diagnostic method with up to 85% positivity in AL amyloidosis, though ATTR amyloidosis could be only detected in 15% of cases [34].
Prostate biopsy	1.4%	Prospective diagnosis of prostate amyloidosis [36].
Carpal tunnel biopsy	10.2%	Samples obtained during carpal tunnel release procedure [29].
Ligamentum flavum biopsy	21.3%–26.5%	Yield represents findings of detectable TTR amyloid on spinal canal stenosis samples [32,38].

TTR, transthyretin.

### 4.1 Orthopedic Manifestations

#### 4.1.1 Carpal Tunnel Syndrome

Patients with transthyretin amyloidosis frequently present with characteristic bilateral carpal tunnel syndrome (CTS), often preceding the onset of symptomatic heart failure. These symptoms along with a noted surgical history of carpal tunnel release have been considered clinical “red flags” among patients with clinical suspicion for CA due to both AL and ATTR [29]. This feature of the disease has been utilized for early intervention for ATTR with targeted screening programs as CTS often manifests 5–10 years prior to cardiac involvement [29,39]. Histologic analysis of amyloid fibril deposited in the carpal tunnel has identified several proteins, including amyloid β-microglobulin, amyloid A, ATTR, and AL. Additionally, amyloid fibrillar deposition has been implicated in other extracardiac musculoskeletal structures, including flexor tendon sheath, fascia, vessel walls, and synovial tissue [40]. These misfolded tetramers deposit in connective tissue as well as endoneurium [41,42].

A history of bilateral CTS as well as multiple carpal tunnel release surgeries among men older than 50 and women older than 60 have been used as screening criteria for cardiac amyloidosis [29]. One study noted that approximately 20.3% of patients with ATTR cardiac amyloidosis presented with a history of CTS, with ATTR amyloidosis patients presenting significantly more frequently than AL amyloidosis patients [43]. A prospective, cross-sectional study of patients undergoing carpal tunnel release surgery demonstrated that 10 of 98 (10.2%) patients had a biopsy of synovial tissue that was positive on Congo red staining for amyloid [29]. In the CACTUS trial, 4.8% of patients screened by intraoperative pathology at the time of carpal tunnel release demonstrated concurrent cardiac amyloidosis with universally low disease severity scores [9]. Similarly, other orthopedic conditions have been strongly associated with primarily ATTR-CM. Surgical procedures such as hip and knee arthroplasty were significantly more common among patients with ATTR-CM (hip arthroplasty: relative risk [RR] 5.61, 95% confidence interval [CI] 3.80–8.29; knee arthroplasty: RR 3.32, 95% CI 2.25–4.64) [44]. In our experience, hand surgery centers without dedicated amyloid centers can utilize pre-established criteria coupled with referral to centers capable of performing the genetic, neurologic, and cardiac evaluations needed for amyloidosis assessment [45].

#### 4.1.2 Lumbar Stenosis

Similarly to CTS, amyloid deposition in the ligamentum flavum (LF) with ensuing hypertrophy and consequential compression of the vertebral canal has been implicated in lumbar stenosis and myelopathy associated with systemic amyloidosis [32,46]. Patients frequently present with neurogenic claudication, distinguished by buttock, thigh, or calf aching, cramping, sensory imbalance, and paresthesia [47]. Symptomatic patients may experience restricted mobility with a cascade of adverse effects [48]. Among a cohort of 56 patients with lumbar spinal canal stenosis, magnetic resonance imaging of LF among patients with ATTR-CM demonstrated that LF thickness as well as lumbar spinal segmental instability was proportional to the amount of transthyretin amyloid deposition [30]. Patients with amyloidosis are at higher risk of recurrent spinal stenosis and may require multiple orthopedic interventions. Like CTS, cardiomyopathy among these patients tends to occur later in their disease course [32].

Comprehensive multidisciplinary collaboration between specialties such as orthopedics and cardiology may help address gaps in the current model. In turn, systematic pathologic tissue screening of excised specimens at the time of noncardiac surgery offers a potentially effective strategy of identifying patients early and facilitating more prompt intervention with disease-modifying therapy.

#### 4.1.3 Biceps Tendon Rupture

One third of patients with ATTR-CM and congestive heart failure have been reported to have asymptomatic or spontaneous distal biceps tendon (DBT) rupture [49]. Data supporting programmatic screening for ATTR-CM with intraoperative tissue sampling among patients with biceps tendon rupture remains limited. Among 53 otherwise asymptomatic patients with DBT ruptures who underwent operative repair at a single center, 5 patients (9.4%) had intraoperative distal biceps biopsies that were positive for amyloid deposition on Congo red staining. These patients had no diagnosis of amyloidosis at the time of orthopedic surgery [50]. In a prospective single-center study, one (3%) of out 30 patients with DBT who underwent operative repair had evidence of amyloid deposition on intraoperative biopsy [51]. Neither of these studies determined the presence of cardiac amyloidosis on follow-up. Though patients with subclinical ATTR-CM and spontaneous DBT ruptures can be identified with tissue screening, yield based on previous experiences appears lower compared to carpal tunnel screening.

### 4.2 Gastrointestinal Manifestations

GI manifestations of ATTR amyloidosis are common; however, clinical symptoms often include non-specific GI complaints, including unintentional weight loss, early satiety, nausea, vomiting, and changes in bowel pattern. In some cases, such symptoms may be secondary to other systemic dysfunction or medication side effects, which can make evaluation and diagnosis challenging [52]. GI involvement can often be subclinical in the early stages of amyloidosis, further contributing to diagnostic delays [53]. While GI symptoms in AL amyloidosis are similar to those of ATTR amyloidosis, GI infiltration is more frequently implicated in systemic AL amyloidosis [54].

The pathophysiology of gastrointestinal amyloidosis involves three main mechanisms: (1) mucosal amyloid deposition which often presents as malabsorption symptoms, such as nausea, vomiting, diarrhea, or protein-losing enteropathy; (2) neuropathic involvement, including deposition within the enteric nervous system, which manifests as dysautonomia and GI dysmotility [53]; and (3) vascular involvement, which can present as GI bleeding or ischemia [52].

The gold standard evaluation of mucosal GI amyloid deposition with endoscopic biopsy with Congo red staining is limited as it cannot identify neuropathic involvement within the GI tract. There is limited data that suggest duodenal biopsies offer the highest diagnostic yield [33]; however, random biopsies targeting any areas of mucosal irregularity or areas corresponding to symptoms remain the most conventional approach [52]. ATTR amyloid deposition usually is found in the muscular and subserosal layers and endoscopically has a granular appearance, in contrast to a mass-like protrusion or friable erosion in inflammatory, neoplastic, or vascular pathologies [33].

Neuropathic involvement of GI amyloid most commonly presents as dysmotility, presenting at various stages within the GI tract from esophageal to intestinal as well as rectal incontinence [53]. Gastric retention is common in transthyretin amyloidosis, with a high prevalence of delayed gastric emptying present even early in disease onset. Some evidence suggests that autonomic neuropathy only weakly correlates with gastric retention [55].

There are also notable differences in GI symptom manifestation between types of amyloidosis, with GI bleeding and ischemia rare in ATTR amyloidosis compared with AL amyloidosis. Thus, there are likely multiple factors (e.g., age, other comorbidities) involved in dysmotility symptoms, and the severity of GI symptoms is likely a poor predictor of true GI dysfunction and amyloid involvement.

The frequency of GI manifestations of ATTR amyloidosis is estimated to be approximately 63%. GI symptoms were more frequently observed in vATTR amyloidosis than wtATTR and most commonly appeared as unintentional weight loss and early satiety, according to the Transthyretin Amyloidosis Outcomes Survey (THAOS). This study longitudinally observed 1579 patients with hereditary transthyretin amyloidosis and 160 patients with wild-type transthyretin amyloidosis [56]. Endoscopically, rates of GI amyloid deposition are identified closer to 40% in retrospective review of histopathological specimens [33,57]. Despite this statistic, prospective tissue screening has not necessarily been successful. Though the small intestine is the most frequently involved site in systemic amyloidosis, studies are divided on its yield, with some reporting duodenal biopsy positivity in up to 100% of patients and others estimating a diagnostic yield of 50–70% [34]. Similarly, rectal biopsy yield varies significantly based on the type of amyloidosis, with up to 85% positivity for AL amyloidosis but only 15% in ATTR amyloidosis [34]. A single center prospective screening study in which gastrointestinal mucosal samples from different GI tract locations were assessed among patients with gastrointestinal symptoms suggestive of systemic amyloidosis was not effective in identifying ATTR patients. Among this cohort of 97 patients, no biopsies had positive Congo red staining [35]. That said, due to the systemic distribution of amyloid, positive biopsy even in the absence of GI symptoms should prompt further investigation.

Treatment of GI amyloidosis remains supportive, such as lifestyle modification and symptom-based medical management. There are currently no medications proven to remove amyloid deposition in GI mucosa or neural circuits [52]. Early multidisciplinary team involvement, including nutritional support and palliative therapies, are recommended to maintain quality of life. Treatments to maintain nutritional status with more novel gene silencing therapies like patisiran (Alnylam Pharmaceuticals, Incorporated, Cambridge, Massachusetts) have shown to improve modified body mass index and symptoms of autonomic neuropathy with treatment compared to placebo, indicating relative reversal of autonomic neuropathy and associated GI symptoms [58,59].

## 5. Utility of Urologic Sampling

Older male patients with wild-type ATTR-CM are also at risk of prostate conditions such as benign prostatic hyperplasia (BPH) or elevated prostate-specific antigen (PSA) levels. Urologic procedures, including prostate biopsy and transurethral resection, offer a unique opportunity for tissue diagnosis. Studies analyzing prostate tissue have identified amyloid deposition in approximately 1.4% of patients, the majority of whom lacked pathogenic ATTR variants upon genetic testing [36]. Of seventeen patients with identified amyloid deposition that pursued echocardiographic evaluation, two met the diagnostic echocardiographic criteria and both were eventually diagnosed with cardiac amyloidosis [36]. Autopsy data have confirmed that prostatic ATTR deposition often correlates with subclinical cardiac involvement at a similar frequency to GI manifestations [60]. While not all patients with BPH or elevated PSA levels will undergo tissue sampling or prostate surgery, the presence of amyloid deposits on intraoperative histology can prompt further cardiac evaluation in unidentified patients.

## 6. Operative Cardiac Pathology

Analysis of tissue from cardiothoracic surgery can provide alternative means of identifying ATTR-CM in patients with pre-existing structural heart disease. As ATTR-CM can be misconstrued as hypertrophic obstructive cardiomyopathy, septal myectomy specimens should routinely be sent to pathology for Congo red staining. A review of a single-center myectomy database found 27 of 3292 (0.8%) reviewed myectomy samples showed cardiac amyloidosis on pathologic examination [37]. Patients with aortic stenosis and ATTR-CM have been shown to have higher rates of mortality and heart failure-related hospitalization than patients with aortic stenosis alone [61]. Systematic non-invasive screening of patients undergoing transcatheter aortic valve replacement (TAVR) has demonstrated variable yield [62]. In patients undergoing surgical aortic valve replacement (AVR), early identification of amyloidosis via histopathological analysis of the explanted native aortic valve can also facilitate early diagnosis.

Among patients with end-stage heart failure requiring a left ventricular assist device (LVAD), the core of myocardium removed to permit the insertion of the LVAD inflow cannula at the apex of the heart can also, in rare cases, be evaluated for amyloid deposits to reveal a diagnosis of ATTR-CM [63]. Overall, intraoperative cardiac pathology is an effective tissue sampling modality that should be considered among patients in whom ATTR-CM is expected and who are undergoing cardiac surgery.

## 7. Treatment and Surveillance Implications for Tissue Screening

Patients identified through tissue screening can fall into several distinct but nuanced treatment pathways provided light-chain amyloidosis has been ruled out: (1) clinical cardiomyopathy with positive scintigraphy, (2) positive scintigraphy without clinical cardiac disease and with normal imaging and biomarkers, (3) imaging or biomarker evidence of infiltrative disease with negative scintigraphy, or (4) negative scintigraphy with normal imaging and biomarkers. Consensus guidance and expert opinion generally support the treatment of those with positive scintigraphy and overt cardiovascular disease, while most would avoid treating those without myocardial uptake, imaging, or laboratory evidence of cardiac involvement [64]. The basis of these recommendations stems from the available clinical trial data that studied the impact of tafamidis in only symptomatic patients (ATTR-ACT Trial) [65]. The more recent HELIOS-B and ATTRIBUTE-CM also demonstrated that vutrisiran (Alnylam Pharmaceuticals, Incorporated, Cambridge, Massachusetts), a ribonucleic acid (RNA) interference agent, and acoramidis (BridgeBio Pharma, Incorporated, Palo Alto, California, USA), a TTR stabilizer, reduced the risk of cardiovascular death and HF hospitalization in patients with elevated NT-proBNP and HF, excluding those with asymptomatic disease [15,66,67].

Therefore, the treatment of positive scintigraphy in the absence of cardiac symptoms, which is not infrequent in the context of programmatic or convenient sampling of extra-cardiac tissue, remains controversial. Concomitant evaluation for AL-CM with ATTR-CM is still recommended though the cascade of testing and appointments that results from incidental monoclonal gammopathy of undetermined (MGUS), which can be present in up to 20% of older adults, is an unintended consequence of comprehensive testing in older adults [1]. Silencer or stabilizer initiation in the presence of proven myocardial uptake based on scintigraphy, early infiltrative disease based on CMR imaging, or asymptomatic elevation in high-sensitivity troponin or NT-proBNP has significant practice variation and remains an area of debate amongst CA experts [64]. Furthermore, payor coverage for these edge cases is variable, with some clinicians feeling that such costly medications should be reserved for trial-based indications.

Importantly cardiac magnetic resonance imaging (CMR), while not considered a confirmatory non-invasive modality, can provide a high-resolution anatomic and functional assessment to differentiate cardiac amyloidosis from other causes of cardiomyopathy and aid in understanding the relative contribution of amyloid deposition to a patient’s cardiac phenotype. Specifically, use of late gadolinium enhancement (LGE) may reveal diffuse subendocardial enhancement, diffuse transmural enhancement, or difficulty nulling of the myocardium, which are a few of the characteristic patterns [2,68]. Indeed, CMR can provide some clarity if nuclear imaging, biomarkers, and clinical symptoms are misaligned in the evaluation tissue screening cases. For example, a patient with elevated biomarkers, negative scintigraphy, some manifestations of cardiac disease (e.g., paroxysmal atrial fibrillation), an infiltrative pattern on CMR suggestive of early CA may push some amyloid experts toward treatment, but little consensus exists in this domain of practice.

The lack of prospective evidence around asymptomatic treatment is counterbalanced by the expected progressive nature of ATTR-CM based on known natural history and trial evidence showing that earlier stage patients with NYHA class I and II disease receive more benefit than those with more symptomatic disease. Additionally, there is longitudinal retrospective evidence that treatment with TTR stabilizers in patients with ATTR-CM but without clinical heart failure at initial evaluation was associated with less progression to clinical heart failure as well as with improved survival over a median follow-up period of 3.7 years [69]. Investigation into early initiation of therapy in patients without overt symptoms of cardiac involvement is ongoing [70]. Lastly, in patients with carpal tunnel pathology with amyloid but no evidence of clinical or imaging-based myocardial involvement, surveillance with interval TTE and/or repeat scintigraphy is reasonable based on expert opinion but has not been prospectively validated. Given the relatively good safety and tolerance profile of stabilizer therapy, many clinicians favor treating early disease if they can receive authorization from the payor. As with all clinical decisions where there is incomplete evidence, shared decision making balancing specific patient factors is recommended.

## 8. Conclusion

Patients with ATTR-CM are often identified late in their disease course despite improved use of non-invasive diagnostics. Both real-world and histopathologic data have suggested that extracardiac manifestations of systemic amyloidosis often precede amyloid heart disease. Multidisciplinary collaboration, incorporating opportunistic screening of specimens at the time of noncardiac and cardiac surgery, offers a potentially effective strategy for identifying patients early in their disease course. Given variable yield and the significant anxiety and cost associated with evaluating and possibly treating asymptomatic patients, refinement of screening criteria and further investigation into early treatment and disease trajectory is needed. At present, many studies do not clearly differentiate between wtATTR and ATTR, which remains an important distinction given heterogeneity and differences in disease courses between the two entities. Treatment of asymptomatic or minimally symptomatic disease is an evolving area without clear consensus or strong evidence to guide management.

Future progress will depend on developing systems of care that operationalize screening in high-risk populations, particularly within noncardiac systems using systemic tissue screening. The ideal yield and cost-effectiveness of such strategies, as well as the optimal timing for intervention in asymptomatic individuals, remain areas for active investigation.
